# 1841. Incidence and Predictors of Complications in Gram Negative Bloodstream Infection

**DOI:** 10.1093/ofid/ofac492.1470

**Published:** 2022-12-15

**Authors:** Utpal Mondal, Erin Warren, Julie Ann Justo, Julie Ann Justo, Joseph Kohn, P Brandon Bookstaver, Majdi N Al-Hasan, Majdi N Al-Hasan

**Affiliations:** University of South Carolina/Prisma Health, Columbia, South Carolina; Prisma Health Richland Hospital, Prosperity, South Carolina; University of South Carolina College of Pharmacy, Columbia, South Carolina; University of South Carolina College of Pharmacy, Columbia, South Carolina; Prisma Health Midlands, Columbia, South Carolina; Prisma Health Richland - University of South Carolina, Columbia, South Carolina; University of South Carolina School of Medicine, Columbia, South Carolina; University of South Carolina School of Medicine, Columbia, South Carolina

## Abstract

**Background:**

The incidence of complications in Gram negative bloodstream infection (GN-BSI) is not clearly defined. This retrospective cohort study evaluates the incidence of complications within 90 days of GN-BSI and examines the predictors for these complications.

**Methods:**

Hospitalized adult patients with monomicrobial GN-BSI at Prisma Health-Midlands hospitals in South Carolina between 1/1/2012 and 6/30/2015 were evaluated. Complications of GN-BSI were defined as endocarditis, septic arthritis, osteomyelitis, spinal infections, deep seated abscesses, and recurrent GN-BSI within 90 days of the initial episode. Clinical and microbiological variables were assessed as potential risk factors for complications, including initial response to antimicrobial therapy within the first 72-96 hours of GN-BSI using the early clinical failure criteria. Kaplan-Meier analysis and multivariate Cox proportional hazards regression were used to examine the incidence and risk factors of complicated GN-BSI, respectively.

**Results:**

A total 752 patients with GN-BSI were included in the study. The median age was 66 years and 380 (50.6%) were women. The urinary tract was the most common source of GN-BSI (378; 50.2%) and *Escherichia coli* was the most common bacteria (375; 49.9%). Overall, 13.9% developed complications within 90 days of GN-BSI. The median time to identification of these complications was 5.2 days from the index GN-BSI (interquartile range 1-28 days). The incidence of complications was notably higher in BSI due to *Serratia* species (39.7%), *Proteus mirabilis* (35.7%), and *Pseudomonas aeruginosa* (21.5%) than other bacteria (11.1%; log-rank p< 0.001). Independent risk factors for complications included early clinical failure criteria, non-urinary source, presence of indwelling prosthetic devices, BSI due to *Serratia* species, *P. mirabilis* or *P. aeruginosa*, and persistent GN-BSI (Table).

**Figure:**
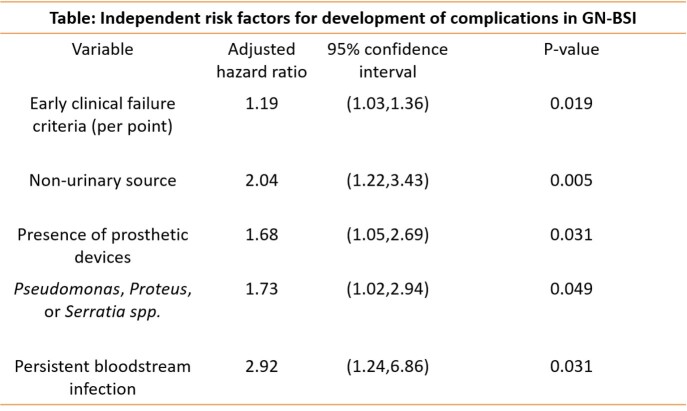

**Conclusion:**

At least 13.9% of patients developed complications within 90 days of GN-BSI. Risk of development of complications may be predicted by specific host and microbiological factors. Stratification of patients based on these risk factors may aid in identifying patients requiring further diagnostic work up for early detection of complications.

**Disclosures:**

**Julie Ann Justo, PharmD, MS, FIDSA, BCPS-AQ ID**, bioMerieux: Honoraria|bioMerieux: Honoraria|Entasis Therapeutics: Advisor/Consultant|Entasis Therapeutics: Advisor/Consultant|Gilead Sciences: Advisor/Consultant|Gilead Sciences: Advisor/Consultant|Merck & Co: Advisor/Consultant|Merck & Co: Advisor/Consultant|Shionogi: Advisor/Consultant|Shionogi Inc.: Advisor/Consultant|Spero Therapeutics: Honoraria|Spero Therapeutics: Honoraria|Vaxart: Stocks/Bonds **Julie Ann Justo, PharmD, MS, FIDSA, BCPS-AQ ID**, bioMerieux: Honoraria|bioMerieux: Honoraria|Entasis Therapeutics: Advisor/Consultant|Entasis Therapeutics: Advisor/Consultant|Gilead Sciences: Advisor/Consultant|Gilead Sciences: Advisor/Consultant|Merck & Co: Advisor/Consultant|Merck & Co: Advisor/Consultant|Shionogi: Advisor/Consultant|Shionogi Inc.: Advisor/Consultant|Spero Therapeutics: Honoraria|Spero Therapeutics: Honoraria|Vaxart: Stocks/Bonds **P. Brandon Bookstaver, PharmD**, Spero Therapeutics: Advisor/Consultant.

